# Parkinson's disease peripheral immune biomarker profile: a multicentre, cross-sectional and longitudinal study

**DOI:** 10.1186/s12974-022-02481-3

**Published:** 2022-05-24

**Authors:** Yuanyuan Li, Yan Yang, Aonan Zhao, Ningdi Luo, Mengyue Niu, Wenyan Kang, Anmu Xie, Hong Lu, Lei Chen, Jun Liu

**Affiliations:** 1 Department of Neurology and Institute of Neurology, Ruijin Hospital Affiliated to Shanghai Jiaotong University School of Medicine, Shanghai, 200025 China; 2 Department of Neurology, The Affiliated Hospital of Jining Medical University, Jining, 272000 China; 3 Department of Neurology, Ruijin Hospital North Affiliated to Shanghai Jiaotong University School of Medicine, Shanghai, 200025 China; 4 Department of Neurology, The Affiliated Hospital of Qingdao University, Qingdao, 266003 China; 5 Department of Neurology, The First Affiliated Hospital of Zhengzhou University, Zhengzhou, 450000 China; 6 Department of Neurology, Tianjin Key Laboratory of Cerebrovascular and Neurodegenerative Diseases, Tianjin Huanhu Hospital, Tianjin, 300350 China; 7 CAS Center for Excellence in Brain Science and Intelligence Technology, Ruijin Hospital Affiliated to Shanghai Jiaotong University School of Medicine, Shanghai, 200025 China

**Keywords:** Chemokines, Cytokines, Multicentre study, Parkinson’s disease, iRBD

## Abstract

**Background:**

Inflammations play crucial role in the pathogenesis of Parkinson’s disease (PD), however, their possible value in the diagnosis or tracking of the progress of PD is still limited, because of discordant results in the literature and a lack of information regarding its reproducibility. Thus, overall longitudinal and cross-sectional studies are needed. This multicentre study was designed to investigate the association between multiple peripheral immune biomarkers and the development and progression of PD.

**Methods:**

This was a longitudinal and multicentre study. First, we measured the levels of five typical cytokines and five focused chemokines in 76 PD patients and 76 healthy controls (HCs) in a discovery cohort. Then, a validation cohort of 80 PD and 80 HC participants was recruited from four multicentre locations. In addition, a prospective follow-up of early-stage PD patients was performed with significant biomarkers. Finally, we performed further verification in an exploratory set of patients with idiopathic REM sleep behaviour disorder (iRBD).

**Results:**

In the discovery set, CXCL12, CX3CL1 and IL-8 levels were significantly higher in PD patients than in HCs (*p* < 0.05). The receiver-operating characteristic (ROC) curve for a combination of these three biomarkers produced a high area under the curve (AUC) of 0.89 (*p* < 0.001). Moreover, four biomarkers (the previous three and CCL15) were significantly associated with PD in the discovery and validation cohorts. Furthermore, in the prospective follow-up cohort, CX3CL1 levels were associated with motor progression after a mean interval of 43 months. In addition, CX3CL1 and IL-8 levels were higher in iRBD patients than in HCs.

**Conclusion:**

We showed a correlation between a profile of four peripheral immune biomarkers and PD development and progression. Our findings may provide a basis whereby PD patients with abnormal inflammatory profiles can be identified and receive timely therapeutic interventions.

**Supplementary Information:**

The online version contains supplementary material available at 10.1186/s12974-022-02481-3.

## Background

Parkinson’s disease (PD) is a neurodegenerative disorder characterized by significant loss of dopaminergic neurons in the substantia nigra. Recently, there has been a large body of research searching for biomarkers useful in the early stages and for the differential diagnosis of PD and many studies are underway to identify disease-specific biomarkers in cerebrospinal fluid (CSF), peripheral plasma and other bodily fluids. Growing evidence has suggested that inflammation plays an important role in the pathologic features and symptoms of PD [1, 2] and inflammatory biomarkers could be potential early indicators of neurodegenerative disease.

Abundant inflammation has been found in several animal models of dopaminergic neuron degeneration and in PD patients, resulting in the impaired regulation of several cytokines and chemokines secreted by activated microglia, which precedes the progression of PD [3]. Inflammatory factors in blood are more readily accessible than those in CSF and the passage of some of these factors through the blood–brain barrier allows them to be measured in the periphery [4]. Moreover, some peripheral inflammatory markers can cross the blood–brain barrier [5]; therefore, peripheral cytokines and chemokines may be useful biomarkers for the detection of PD progression.

The inflammatory cytokine and chemokine levels in the plasma and serum of PD patients vary significantly. Several studies have shown that PD pathophysiology is associated with increased inflammatory status [6]. However, discordant results in the literature and a lack of information regarding the stability of inflammatory factors in different cohorts have hampered progress. To fill this void, we measured 10 promising cytokines and chemokines in a discovery cohort, validated them in four validation sets and further verified them in an exploratory set of patients with idiopathic REM sleep behaviour disorder (iRBD). Moreover, we evaluated the potential of longitudinal factors as prognostic biomarkers for monitoring PD progression. We hypothesized that the combination of certain biomarkers would help with the early diagnosis of PD and tracking its progression.

## Methods

### Study population

For the discovery cohort, 76 PD patients and 76 age- and sex-matched healthy controls (HCs) were recruited from the Department of Neurology, Ruijin Hospital Affiliated with Shanghai Jiaotong University School of Medicine from 2017 to 2019. The diagnosis of PD was performed according to the International Parkinson and Movement Disorder Society (MDS) diagnostic criteria by at least two neurologists skilled in movement disorders [7]. For the validation set, 80 PD patients and 80 matched controls from four medical centres, including Tianjin Huanhu Hospital, the Affiliated Hospital of Qingdao University, the Affiliated Hospital of Jining Medical University and the First Affiliated Hospital of Zhengzhou University, were enrolled from 2018 to 2019. All of the participants enrolled form five centers were Han Chinese people without family history, which reduced the ethnical genetic variation between groups. In addition, 39 PD patients in the early stage from the discovery cohort were chosen for follow-up assessments. This study was approved by the ethics committee of Ruijin Hospital, Shanghai Jiaotong University School of Medicine. Subjects with any peripheral inflammatory diseases or taking anti-inflammatory medicines were excluded from the cohort.

As an exploratory set, 45 iRBD patients with video-polysomnography confirmation were subsequently recruited based on the consensus criteria of the International RBD Study Group [8]. All iRBD patients were examined by neurologists to exclude motor signs of parkinsonism or secondary causes. Thirty-five controls without neurological disorders were also enrolled. The ages of all participants ranged from 50 to 80 years, and patients taking anti-inflammatory medicines were excluded. All participants provided written informed consent to the research protocol, which was approved by the local medical ethical committee and conformed to the Helsinki declaration.

### Demographics and clinical assessment of PD patients

For the PD patients, the disease stage was determined using the modified Hoehn and Yahr (H-Y) staging scale (range 0–5) [9]. The motor subscale of the Unified Parkinson's Disease Rating Scale (UPDRS) was used to evaluate motor symptoms in the ON medication state. The Non-Motor Symptom Questionnaire (NMSQ), Scale for Outcomes in PD-Autonomic (SCOPA-AUT), Sniffin’ Sticks 16-item test (SS-16), 17-item Hamilton Depression Rating Scale (HAMD-17), Mini-Mental State Examination (MMSE), Montreal Cognitive Assessment (MoCA) and Sleep Behavior Disorder Screening Questionnaire (RBDSQ) were also completed.

### Sampling and biomarker selection

Whole blood samples (5 mL) were collected in EDTA tubes and centrifuged at 3000 rpm for 15 min at room temperature. Plasma samples were frozen within 2 h of collection and stored at − 80 °C until assayed. All the blood from five centres was obtained simultaneously with the clinical information accessed. Ten inflammatory cytokines and chemokines (IL-8, TNF-α, TGF-β, IL-10, IL-6, CXCL12, CX3CL1, CCL15, CCL3 and CCL20) were included based on a review of the existing literature [10, 11] and their levels were measured using a Meso Scale Discovery (MSD) assay. Selection was based on the following criteria: (1) inflammatory chemokines or cytokines had a plausible association with PD-related phenotypes as previously reported [11, 12] or based on our previous work [13]; (2) all of the measurements were within the limit of detection in a pilot project using MSD U-PLEX Human Biomarkers kits.

### MSD assay

The inflammatory markers from each of the participating centres were all measured using a human multiple-chemokine panel kit (MSD, Rockville, MD, USA) in accordance with the manufacturer’s instructions. For each marker, the concentrations were calculated with reference to a standard curve derived using various concentrations of the standards assayed in the same manner as the cell culture supernatants. The lower limit of detection (LLOD) was calculated as a signal concentration that was 2.5 SD greater than the zero calibrator. The upper limit of detection was calculated as a signal concentration that was 2.5 SD below the upper plateau of the standard curve. To avoid potential cross-interactions among different antibodies, not all ten markers were measured simultaneously. CX3CL1, IL-10, IL-6, IL-8, CCL3, CCL20 and TNF-α were measured simultaneously in one MSD assay, while TGF-β, CXCL12 and CCL15 were measured individually in three separate assays according to the manufacturer's guidance. All plasma samples were assayed in duplicate, and the results were averaged for analysis.

### Statistical analysis

The data were analysed by SPSS software version 20.0 (SPSS Inc., Chicago, IL, USA). Owing to the non-normal distribution of the ten markers and clinical characteristics, Mann–Whitney *U* test was performed to analyse the difference between HC and PD participants. Statistical significance was established as a two-sided *p* value < 0.05, and multiple testing was controlled by Bonferroni correction (*p* < 0.05/10 biomarkers). Conditional logistic regression analysis was used to quantify the association of inflammatory biomarkers with PD adjusted for age, sex and sites, and the effect-size estimates were expressed as odds ratio (OR) and 95% confidence intervals (CIs). In addition, the association of inflammatory biomarkers with sex and age (≤ 60 or > 60) was separately analysed. Inflammatory biomarker relationships were assessed by Pearson correlation analyses. A receiver-operating characteristic (ROC) curve was created to evaluate the role of inflammatory biomarkers in the diagnosis of PD. For the longitudinal analysis, the increase or decrease of cytokines were defined by the changes of the value between baseline and follow-up, the “increase” group indicates difference above 0, while “decrease” means less or equal to 0. To assess associations between longitudinal chemokine levels and progression outcomes, Cox proportional hazards regression models were used to estimate hazard ratio (HR) with 95% CIs adjusted for age and sex.

## Results

### Characteristics of the participants

A total of 76 PD and 76 HC participants were enrolled in the discovery cohort, and the characteristics and clinical features (disease duration and UPDRS scores) are given in Table 1. The mean age of the participants was 62.2 ± 7.5 years in the PD group and 60.2 ± 8.2 years in the HC group, and no significant differences in sex were found. For the validation group, the characteristics and clinical features of the participants were obtained from four multicentre locations, with the sex and age distributions matched.

**Table 1:** Baseline and clinical characteristics of HC and PD participants

Characteristics	Discovery cohort			Validation cohort			*P*^a^	*P*^b^
	PD^a^ (*N* = 76)	HC^b^ (*N* = 76)	*p*	PD^a^ (*N* = 80)	HC^b^ (*N* = 80)	*p*		
Age (years)	62.2 ± 7.5	60.2 ± 8.2	0.14	63.7 ± 8.5	64.9 ± 8.8	0.40	0.25	0.01
Sex, N								
Female	38	28	0.14	44	39	0.43	0.63	0.15
Male	38	48		36	41			
Disease duration (years)	4.9 ± 4.3	/		3.6 ± 3.6	/			
UPDRS I	8.0 ± 6.1	/	/	6.2 ± 4.5	/	/	0.05	/
UPDRS II	11.5 ± 6.8	/	/	12.7 ± 6.9	/	/	0.28	/
UPDRS III	28.4 ± 15.1	/	/	35.4 ± 18	/	/	0.01	/

Data are expressed as mean and standard deviation (SD), as appropriate

P^a^: comparation of PD participants between discovery and validation cohort; P^b^: comparation of HC participants between discovery and validation cohort

*PD* Parkinson’s disease, *UPDRS* Unified Parkinson's Disease Rating Scale

### Distribution of the potential markers in the discovery set

**Fig. 1:**
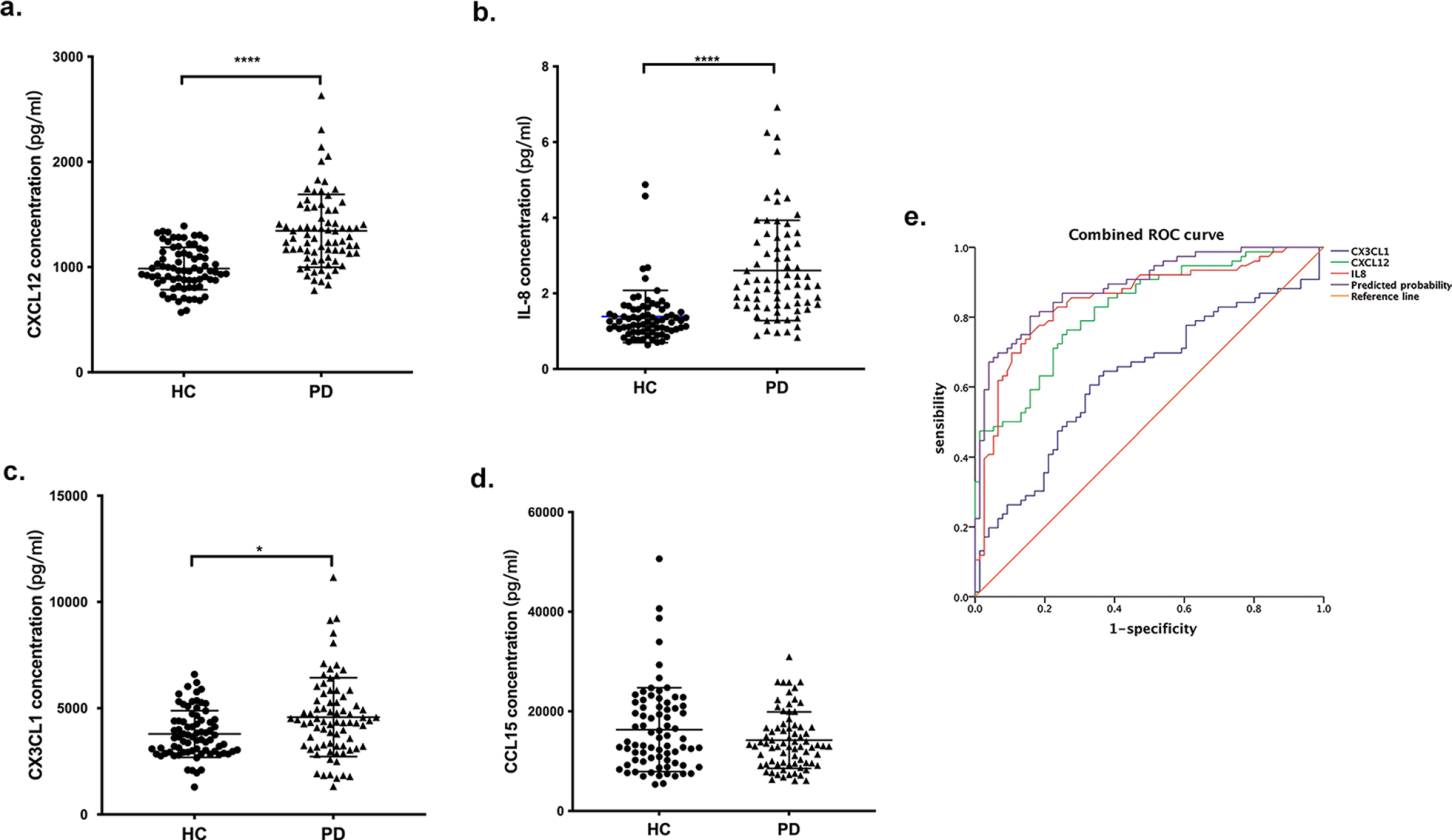
Distribution of CXCL12, IL-8, CX3CL1 and CCL15 between HC and PD participants and ROC curve analysis. **a**–**d** Levels of CXCL12, IL-8, CX3CL1 and CCL15 in plasma between two groups measured by MSD assay. **e** ROC curve between two groups. The blue line is the ROC curve for CX3CL1, and the AUC is 0.63. The green line is the ROC curve for CXCL12, and the AUC is 0.83. The red line is the ROC curve for IL-8, and the AUC is 0.85. The purple line is the ROC curve for the integrative model, and the AUC is 0.89. *p < 0.05, ****p < 0.0001

The plasma levels of 10 inflammatory markers in the PD and HC participants in the discovery cohort are shown in Additional file 1: Table S1. Levels of CXCL12 were significantly higher in the PD participants than in the HCs (1344.2 ± 356.3 pg/mL vs. 986.0 ± 201.2 pg/mL, respectively, *p* < 0.001; Fig. 1a). In addition, IL-8 and CX3CL1 levels in the plasma were also higher in the participants with PD (IL-8: 4.4 ± 10.9 vs. 1.4 ± 0.7 pg/mL, *p* < 0.001; CX3CL1: 4768.9 ± 2570.7 vs. 3913.2 ± 1571.6 pg/mL, *p* = 0.006; Fig. 1b, c). No significant differences in the other markers were found between the two groups. In addition, analysis of the relationship between these presented markers and PD disease severity (H-Y stage and UPDRS III) was performed, and no statistical association was obtained (data not shown). As antiparkinsonian drugs may potentially affect the levels of peripheral inflammatory factors, we performed linear correlation analysis, and no correlations were found between levodopa-equivalent daily dose (LEDD) and levels of CXCL12, IL-8, CCL15 or CX3CL1 (*p* = 0.22, 0.13, 0.09 and 0.10, respectively; data not shown).

Based on the marker distribution differences between the PD and HC participants, CXCL12, CX3CL1 and IL-8 were selected for further analysis. Subsequently, a ROC curve analysis was performed to evaluate the discriminatory power of these three markers, both individually and in combination (Fig. 1e). For CXCL12, the AUC was 0.83 with a cutoff value of 1051 pg/mL (*p* < 0.001); for CX3CL1, the AUC was 0.63 with a cutoff value of 3966.9 pg/mL (*p* = 0.006); and for IL-8, the AUC was 0.85 with a cutoff value of 1.7 pg/mL (*p* < 0.001). Moreover, the combination of all significant biomarkers resulted in a significant increase in the C-statistic of 0.89 (95% CI: 0.84 to 0.94, *p* < 0.001), which indicated that the predictive value of the combined biomarker assessment was greater than that of the individual biomarkers (Additional file 1: Table S2).

### Candidate predictors and association of PD in the discovery and validation sets

To assess how these candidate biomarkers stratify PD, we built a logistic regression model of the ten markers, controlling for age and sex. Four of the ten markers were significantly associated with PD, and this association remained significant after the Bonferroni correction for multiple comparisons (*p* < 0.05/10, here 10 is the total number of examined inflammatory biomarkers; Table 2). For the comparison of the above and below cutoff values, the OR (95% CI) was 9 (4.3–18.7) for CXCL12 (*p* = 4.5 × 10^–9^), 3.1 (1.6–6.0) for CX3CL1 (*p* = 7.8 × 10^–4^), 19.0 (8.2–44.3) for IL-8 (*p* = 7.6 × 10^–12^) and 0.3 (0.2–0.7) for CCL15 (*p* = 4.7 × 10^–3^).

**Table 2:** Risk prediction of four potential biomarkers for PD patients in discovery and validation group

Biomarkers	Discovery		Validation		Combined-analysis	
	OR (95% CI)	*p* value	OR (95% CI)	*p* value	OR (95% CI)	*p* value
CXCL12	9 (4.3–18.7)	**4.5 × 10**^**–9**^	2.3 (1.1–4.6)	**0.02**	3.8 (2.4–6.2)	**4.7 × 10**^**–8**^
CX3CL1	3.1 (1.6–6.0)	**7.8 × 10**^**–4**^	2.8 (1.4–5.4)	**2.4 × 10**^**–3**^	2.9 (1.7–4.9)	**1.5 × 10**^**–4**^
IL-8	19.0 (8.2–44.3)	**7.6 × 10**^**–12**^	57.0 (18.5–174.7)	**1.9 × 10**^**–12**^	26.9 (12.9–56.4)	**2.3 × 10**^**–18**^
CCL15	0.3 (0.2–0.7)	**4.7 × 10**^**–3**^	0.5 (0.2–1.0)	**0.045**	0.4 (0.3–0.7)	**0.001**

*p* value was adjusted for age, gender and site under logistic regression analysis model

The bold emphasis in the table means *p* < 0.05

*OR* odds ratio, *95% CI* 95% confidence interval

Furthermore, these four candidate markers were revalidated in an independent cohort from four multicentre locations, and distribution of clinical information among five centers was shown in Additional file 1: Table S3, and no statistic difference was found among five centers on disease duration or HY stage of PD. Significance persisted after validation, the logistic analysis showed that higher levels of CXCL12, CX3CL1, IL-8 and CCL15 were associated with the diagnosis of PD (OR = 2.3 for CXCL12, 2.8 for CX3CL1, 57.0 for IL-8 and 0.5 for CCL15). (Table 2). Moreover, consistent results were shown in a combined cohort of the discovery and validation sets, which confirmed that these four biomarkers were independently associated with PD. Similarly, the distribution levels of CX3CL1, IL-8 in PD patients were higher in PD patients compared with HC participants (*p* = 0.016 and *p* = 0.003, respectively; Additional file 1: Table S4).

### Validation of the significant inflammatory biomarkers in an exploratory set of iRBD patients

**Fig. 2:**
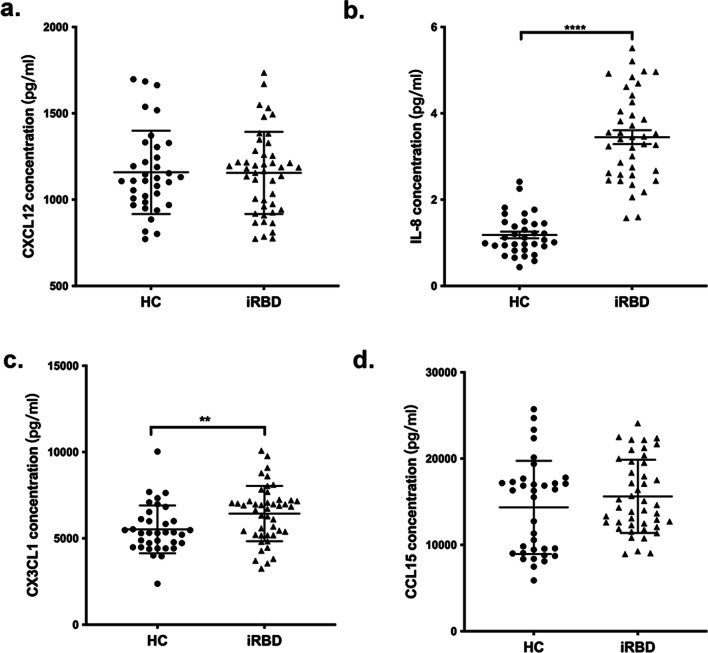
Distribution of CXCL12, IL-8, CX3CL1 and CCL15 between HC and iRBD participants. **a** Levels of CXCL12 between HC and iRBD participants measured by MSD assay. **b** Levels of IL-8 between HC and iRBD participants measured by MSD assay. **c** Levels of CX3CL1 between HC and iRBD participants measured by MSD assay. **d** Between HC and iRBD participants measured by MSD assay.**p < 0.01, ****p < 0.0001

iRBD is a prodromal condition of parkinsonism that is associated with PD [14]. We subsequently compared the levels of these four markers between the iRBD and HC participants. The results showed that the levels of CX3CL1 and IL-8 in plasma were significantly higher in patients with iRBD than in HCs (*p* = 0.009 for CX3CL1; *p* < 0.001 for IL-8; Table 3 and Fig. 2). However, no significant differences were obtained in the distributions of CXCL12 and CCL15 between the two groups.

**Table 3:** Levels of the inflammatory biomarkers in iRBD and HC participants

Biomarkers (pg/mL)	HC (*N* = 35)	iRBD (*N* = 45)	*p*
CXCL12	1158.4 (241.6)	1154.9 (238.19)	0.79
CX3CL1	5522.9 (1389.1)	6435.2 (1603.7)	**0.008**
IL-8	1.2 (0.46)	3.4 (0.16)	** < 0.001**
CCL15	14,344.1 (5403.9)	16,039.7 (5015.2)	0.14

Data are expressed as mean and standard deviation (SD), as appropriate

The bold emphasis in the table means p < 0.05

*iRBD* idiopathic REM sleep behavior disorder

### Longitudinal evaluation of candidate biomarkers and PD progression

**Fig. 3:**
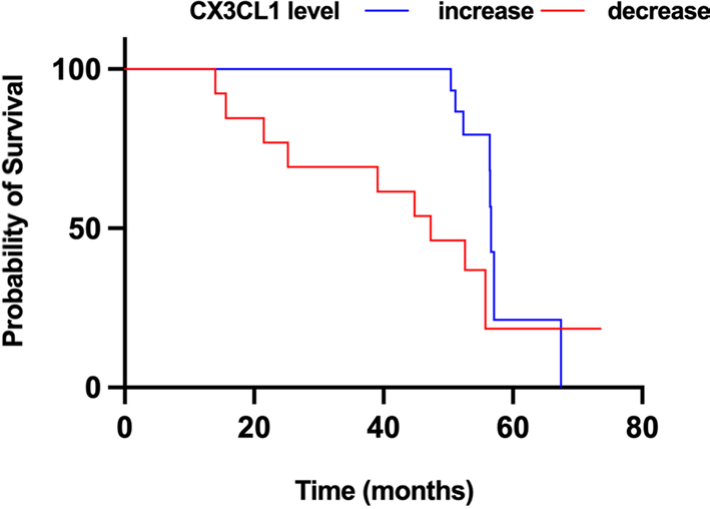
Longitudinal evaluation of CX3CL1 and PD motor-progression. Kaplan–Meier plot of disease-free survival of patients, stratified according to CX3CL1 level

Furthermore, to examine whether these candidate markers could be useful for assessing disease severity, a prospective follow-up of 39 PD patients from the discovery set was conducted, and motor phenotype (UPDRS I-III) and non-motor phenotype (NMSQ) scores and their statistical distributions are shown in Table 4. A change of over 2.3 points on the UPDRS III motor score represents a large clinically meaningful difference [15]. We thus defined motor progression as an 8-point increase in UPDRS III scores (mean of 2.3 points per year) based on a mean follow-up period of 43 ± 2 months and split the longitudinal cohort into 2 subgroups (progression and non-progression) as we did in our previous study [16]. Cox proportional hazards regression Kaplan–Meier analyses showed that decreased levels of CX3CL1 were associated with motor progression (HR = 2.86, *p* = 0.036; Table 5 and Fig. 3).

**Table 4:** Longitudinal assessments in follow-up early PD patients

Characteristics	Baseline (*n* = 39)	Follow-up (*n* = 39)	*p* value
Age (years)	68.5 ± 7.7	/	/
Sex, *N*			/
Male	26	/	/
Female	13	/	/
Disease duration (years)			
UPDRS I	3.3 ± 2.7	6.3 ± 4.1	< 0.001^***^
UPDRS II	9.9 ± 4.3	12.1 ± 4.8	**0.041**
UPDRS III	18.8 ± 8.5	26.9 ± 9.6	< 0.001^***^
NMSQ	8.2 ± 4.1	10.6 ± 4.3	**0.014**
SS-16	6.7 ± 3.3	5.6 ± 2.5	0.099
RBDSQ	5.0 ± 4.3	5.7 ± 3.6	0.45
HAMD-17	5.7 ± 4.4	6.1 ± 4.7	0.71
SCOPA-AUT	13.8 ± 9.6	16.1 ± 8.5	0.27
MMSE	27.2 ± 3.5	27.5 ± 2.8	0.60
MoCA	23.2 ± 5.2	22.8 ± 5.4	0.76

Data are expressed as mean and standard deviation (SD), as appropriate

The bold emphasis in the table means *p* < 0.05

*NMSQ* the Non-Motor Symptom Questionnaire, *SCOPA-AUT* Scale for Outcomes in PD-Autonomic, *SS-16* Sniffin’ Sticks 16-item test, *HAMD-17* 17-item Hamilton Depression Rating Scale, *MMSE* Mini-Mental State Examination, *MoCA* Montreal Cognitive Assessment, *iRBD* idiopathic REM sleep behavior disorder

**Table 5:** Evaluation of four biomarkers for PD motor progression

	Progression (*n* = 17)	Non-progression (*n* = 22)	HR (95% CI)	*p* value
Age (y)	68.0 ± 6.7	68.8 ± 8.5	/	0.75
Sex, *N*				
Male	12	14	/	0.74
Female	5	8		
*CX3CL1*				
Decreased	9	4	2.86 (1.08–7.69)	**0.036**
Increased	8	18		
*IL-8*				
Increased	7	13	0.44 (0.16–1.88)	0.10
Decreased	10	9		
*CXCL12*				
Increased	2	7	0.53 (0.12–2.34)	0.40
Decreased	15	15		
*MIP-5*				
Increased	12	19	1.09 (0.37–3.24)	0.88
Decreased	7	3		

Cox proportional hazards regression models were used to estimate HRs, with 95% CI adjusted for age and sex

The bold emphasis in the table means *p* < 0.05

*HR* hazard ratio, *95 CI* 95 confidence interval

In terms of non-motor symptoms, including NMSQ, MMSE, SCOPA-AUT, SS-16 and RBDSQ, Cox regression analysis were also performed in the longitudinal cohort. Interestingly, in the analysis of progression on cognitive impairment, we found that an increase in CCL15 levels was associated with an increased MMSE score (HR = 7.9, *p* = 0.045; Additional file 1: Table S5). However, no association was found among these four biomarkers and the other clinical characteristic (Additional file 1: Tables S6–S9).

## Discussion

In the present study, we found a strong correlation between a panel of immune-cytokines and chemokine in the peripheral blood and the risk of PD. Furthermore, in multivariate analyses, increased levels of CXCL12, CX3CL1 and IL-8 and a decreased level of CCL15 were all independent diagnostic biomarkers of PD. In addition, the levels of CX3CL1 and IL-8 showed a higher distribution in the exploratory set of iRBD patients. Furthermore, in a prospective follow-up of early-stage PD patients, we found that decreased CX3CL1 levels were associated with motor progression after a mean interval of 43 months.

Several studies have shown that inflammatory biomarkers, including cytokines and chemokines, could be used to help diagnose PD [11, 17, 18]. The levels of proinflammatory cytokines, mainly TNF-α, IL-1β, IL-6, IL-10 and IL-8, and growth factors (EGF and TGF-β1) have been demonstrated by many investigators to be markedly altered in parkinsonian patients when compared with control subjects. Chemokines, such as CXCL12, CCL2, CCL5, CX3CL1 and CCL5, play well-established roles in the immune system, and several recent reports have suggested that chemokines and their receptors may also play a role in the central nervous system (CNS) [19, 20]. Although a number of previous studies have examined the distribution differences of inflammatory biomarkers between PD and HC subjects, no study has focused on the association between these candidate markers and PD susceptibility in a multicentre cohort. Comparing the previous studies, we identified in an exploration cohort out of a bigger panel of cytokines/chemokine, which are validated in a validation cohort using MSD assay. Therefore, the present study increases the strength of the evidence.

CX3CL1 is one of the most important mediators of the communication between neurons and microglia, and its emerging role in PD has been increasingly recognized. Studies show that CX3CL1 plays a neuroprotective role in 6-OHDA-induced dopaminergic lesion, thus it might be an effective therapeutic target for PD [21]. In a PD mouse model, decreased levels of CX3CL1 in the brains of A53T mice were observed, but elevated levels were observed in the peripheral blood [22]. Moreover, Jing Zhang’s team measured the levels of CX3CL1 in the CSF, and no difference was found between PD and control subjects; however, CSF CX3CL1/Aβ_1-42_ was found to be positively correlated with PD severity and progression [23]. In our current cohort, the levels of CX3CL1 were also markedly elevated in PD in independent groups. However, decreased levels of CX3CL1 were associated with the motor progression of PD patients. We supposed this may be due to the fact that CX3CL1 levels are increased in the explorative cohort as a reflection of an anti-inflammatory protective effect, thus it is reasonable that there is an elevated trend in the early stages of PD and a decrease as the disease progresses. This is similar to the distribution in AD studies, as higher CX3CL1 plasma levels were observed in patients with mild to moderate AD than in patients with severe AD [24]. Larger studies are needed to investigate the biomarker potential of CX3CL1 for the diagnosis and prognosis of PD.

The relationship between CXCL12 and PD was first reported by Mika Shimoji based on the data from the postmortem brains of PD patients and control individuals, and it was suggested that CXCL12 may participate in the aetiology of PD [25]. In our previous study, CXCL12 was involved in α-synuclein-triggered neuroinflammation and could be a novel target for the prevention of α-synuclein-triggered ongoing microglial responses [13]. In a small sample, Vahid Bagheri’s team demonstrated that CXCL12 serum levels were significantly elevated in patients with PD compared to controls (30 PD patients and 40 controls) [26]. In our current study, our findings also support the hypothesis that CXCL12 is a candidate biomarker for the diagnosis of PD. Patients in the discovery and validation sets with high levels of CXCL12 were more than 9 and 2.4 times more likely to experience PD, respectively, than those with low CXCL12 levels. The biological mechanisms underlying the role of CXCL12 are not fully understood, and further functional characterization is encouraged.

The trend of IL-8 levels in the peripheral blood of PD patients has not been confirmed. Reale et al. found that the level of IL-8 in PD patients was significantly higher than that in HCs [27]; however, Vineeta’s team found that the level of IL-8 was significantly reduced in the serum of patients with PD [28]. Extending the results of previous studies, our findings demonstrated a strong correlation between the levels of IL-8 and PD. Moreover, regarding CCL15, Hochstrasser et al. showed that in monocytes, CCL15 levels were significantly lower in AD patients than in healthy subjects, but they were increased in AD patient plasma [29]. However, CCL15 plasma levels have been shown to be reduced in AD patients as compared to controls [30]. In our current study, reduced CCL15 levels were strongly associated with PD, which first showed evidence of the value of CCL15 in diagnosing PD.

Dopaminergic therapy influences plasma protein levels; however, according to a recent study that analysed > 1000 plasma proteins in ~ 500 individuals, CXCL12, CX3CL1, CCL15 and IL-8 are not affected by dopaminergic medications [31]. Moreover, no correlations were found between LEDD and levels of these four biomarkers as correlation analysis shown, which strength their predict value.

In summary, we used a combined statistical analysis of CXCL12, CX3CL1 and IL-8 levels to differentiate PD patients from HCs with high sensitivity and specificity. Considering inconsistent results have been reported for individual cytokines and between studies in the literature, our results provide evidence of a heightened proinflammatory cytokine profile in PD patients, strengthening the clinical evidence that patients with PD have an increased inflammatory response.

Our study is not without limitations. On the one hand, our findings need to be replicated in a larger population to further confirm the efficacy of CXCL12, CX3CL1, IL-8 and CCL15 in diagnosing PD. On the other hand, patients enrolled in the present study were not classified by different disease stages or phenotypes, and future studies with large samples specifically designed to examine the association of the four inflammatory biomarkers with different phenotypes of PD are warranted.

## Conclusion

In conclusion, in our current study, we found that certain peripheral immune biomarkers are significant independent predictors of PD development and progression.

## Supplementary Information

**Additional file 1: Table S1**. Plasma levels of 10 inflammatory markers of the discovery cohort. **Table S2**. The ability of combined discovery markers for the diagnosis of PD. Table S3. Clinical characteristic PD participants from five centers. **Table S4**. Plasma levels of four inflammatory markers of the validation cohort. **Table S5**. Evaluation of four biomarkers for progression of MMSE. **Table S6**. Evaluation of four biomarkers for progression of NMSQ. **Table S7**. Evaluation of four biomarkers for progression of SCOPA-AUT. **Table S8**. Evaluation of four biomarkers for progression of SS-16. **Table S9**. Evaluation of four biomarkers for progression of RBDSQ.

## Data Availability

All data generated or analysed during this study are included in this published article.

## Abbreviations

PD: Parkinson’s disease
RBD: Idiopathic REM sleep behaviour disorder
LEDD: Levodopa-equivalent daily dose
MSD: Meso Scale Discovery
CSF: Cerebrospinal fluid
MDS: Movement Disorder Society
UPDRS: Unified Parkinson’s Disease Rating Scale
LLOD: Lower limit of detection
OR: Odds ratio
HR: Hazard ratio
CI: Confidence interval
ROC: Receiver-operating characteristic
CNS: Central nervous system
