# Supplementary material for: Parkinson's disease peripheral immune biomarker profile: a multicentre, cross-sectional and longitudinal study
Source: J Neuroinflammation. 2022 May 24;19:116. doi: 10.1186/s12974-022-02481-3 (PMC9131564; doi:10.1186/s12974-022-02481-3)
Supplement: Supplementary file 1 — Additional file 1: Table S1. Plasma levels of 10 inflammatory markers of the discovery cohort. Table S2. The ability of combined discovery markers for the diagnosis of PD. Table S3. Clinical characteristic PD participants from five centers. Table S4. Plasma levels of four inflammatory markers of the validation cohort. Table S5. Evaluation of four biomarkers for progression of MMSE. Table S6. Evaluation of four biomarkers for progression of NMSQ. Table S7. Evaluation of four biomarkers for progression of SCOPA-AUT. Table S8. Evaluation of four biomarkers for progression of SS-16. Table S9. Evaluation of four biomarkers for progression of RBDSQ. [file 12974_2022_2481_MOESM1_ESM.docx]

**Table S1. Plasma levels of 10 inflammatory markers of the discovery cohort**

| **Biomarkers (pg/mL)** | **HC (N=76)** | **PD (N=76)** | **P value** |
| --- | --- | --- | --- |
| CXCL12 | 986.0(201.2) | 1344.2(356.3) | **<0.001** |
| CX3CL1 | 3913.2 (1571.6) | 4768.9 (2570.7) | **0.006** |
| IL-8 | 1.4 (0.7) | 4.4 (10.9) | **<0.001** |
| CCL15 | 16337.7 (8419.7) | 14212.0 (5679.7) | 0.26 |
| CCL3 | 15.0 (2.9) | 14.7 (2.8) | 0.71 |
| CCL20 | 15.8 (17.6) | 13.4 (6.5) | 0.49 |
| TNF-α | 4.3 (1.8) | 4.1 (1.5) | 0.80 |
| TGF-β | 4265.2.0 (5230.4) | 5616.2 (6999.9) | 0.22 |
| IL-10 | 0.6 (0.2) | 0.6 (0.3) | 0.96 |
| IL-6 | 1.3 (0.4) | 1.8 (2.8) | 0.33 |

Data are expressed as mean and standard deviation.

The bold emphasis in the table means *p* < 0.05.

**Table S2. The ability of combined discovery markers for the diagnosis of PD**

|  | **Cutoff value (pg/ml)** | **Sensitivity** | **Specificity** | **AUC** | **p value** |
| --- | --- | --- | --- | --- | --- |
| CXCL12 | 1051.0 | 82.9 | 65.8 | 0.83 | **<0.001** |
| CX3CL1 | 3966.9 | 64.5 | 63.2 | 0.63 | **0.006** |
| IL-8 | 1.7 | 73.7 | 85.5 | 0.85 | **<0.001** |
| Combined markers | / | / | / | 0.89 | **<0.001** |

**Table S3. Clinical characteristic PD participants from five centers**

| **Clinical Characteristics** | **Discovery**  **site**  **(N=76)** | **Validation site 1**  **(N=17)** | **Validation site 2**  **(N=22)** | **Validation site 3**  **(N=16)** | | **Validation site 4**  **(N=25)** | **P**  **Value** |
| --- | --- | --- | --- | --- | --- | --- | --- |
| Age (years) | 62.2 ± 7.5 | 63.7 ± 7.1 | 63.7 ± 8.7 | 68.8 ± 7.1 | 61.8 ± 7.1 | | 0.039 |
| Sex, N |  |  |  |  |  | |  |
| Female | 38 | 5 | 15 | 9 | 15 | |  |
| Male | 38 | 12 | 7 | 7 | 10 | |  |
| PD Duration (years) | 4.9 ± 4.3 | 2.1 ± 2.0 | 4.2 ± 3.5 | 3.4 ± 2.8 | 4.0 ± 4.1 | | 0.079 |
| HY stage | 2.0 ± 0.9 | 2.2 ± 0.6 | 2.3 ± 0.9 | 1.9 ± 0.8 | 2.5 ± 0.7 | | 0.15 |

Data are expressed as mean and standard deviation (SD), as appropriate.

**Table S4. Plasma levels of four inflammatory markers of the validation cohort**

| **Biomarkers (pg/mL)** | **HC (N=76)** | **PD (N=76)** | **P value** |
| --- | --- | --- | --- |
| CXCL12 | 935.4(204.8) | 990.1(305.3) | 0.19 |
| CX3CL1 | 5624.3 (1245.1) | 6166.7 (1533.4) | **0.016** |
| IL-8 | 2.2 (1.0) | 7.3 (15.6) | **0.003** |
| CCL15 | 13830.0 (6144.4.7) | 13125.1(5835.7) | 0.46 |

Data are expressed as mean and standard deviation.

The bold emphasis in the table means *p* < 0.05.

**Table S5. Evaluation of four biomarkers for progression of MMSE**

|  | **Progression**  **(n=22)** | **Non-progression**  **(n=17)** | **HR**  **(95%CI)** | **p value** |
| --- | --- | --- | --- | --- |
| Age (y) | 69.2 ± 7.6 | 67.5 ± 7.9 | / | 0.49 |
| Sex, N |  | | | |
| Male | 14 | 12 | / | 0.65 |
| Female | 8 | 5 |  |  |
| ***CX3CL1*** |  | | | |
| Increased | 14 | 12 | 0.69  (0.29-1.67) | 0.41 |
| Decreased | 8 | 5 |  |  |
| ***IL-8*** |  | | | |
| Increased | 12 | 8 | 0.94  (0.40-2.22) | 0.89 |
| Decreased | 10 | 9 |  |  |
| ***CXCL12*** |  | | | |
| Increased | 4 | 5 | 0.95  (0.33-2.74) | 0.92 |
| Decreased | 18 | 12 |  |  |
| ***CCL15*** |  | | | |
| Increased | 20 | 11 | 7.9  (1.1-59.1) | **0.045** |
| Decreased | 2 | 6 |  |  |

Cox proportional hazards regression models were used to estimate HRs, with 95% CI adjusted for age and sex.

The bold emphasis in the table means *p* < 0.05.

Abbreviations: HR: hazard ratio; 95% CI: 95% confidence interval.

**Table S6. Evaluation of four biomarkers for progression of NMSQ**

|  | **Progression**  **(n=32)** | **Non-progression**  **(n=7)** | **HR**  **(95%CI)** | **p value** |
| --- | --- | --- | --- | --- |
| Age (y) | 70.0 ± 7.5 | 70.7 ± 8.6 | / | 0.40 |
| Sex, N |  | | | |
| Male | 12 | 6 | / | 0.44 |
| Female | 5 | 1 |  |  |
| ***CX3CL1*** |  | | | |
| Increased | 21 | 4 | 0.73  (0.34-1.58) | 0.42 |
| Decreased | 11 | 18 |  |  |
| ***IL-8*** |  | | | |
| Increased | 16 | 4 | 0.70  (0.34-1.46) | 0.34 |
| Decreased | 16 | 3 |  |  |
| ***CXCL12*** |  | | | |
| Increased | 9 | 0 | 1.21  (0.55-2.69) | 0.63 |
| Decreased | 23 | 7 |  |  |
| ***CCL15*** |  | | | |
| Increased | 25 | 6 | 1.46  (0.62-3.44) | 0.39 |
| Decreased | 7 | 1 |  |  |

Cox proportional hazards regression models were used to estimate HRs, with 95% CI adjusted for age and sex.

The bold emphasis in the table means *p* < 0.05.

Abbreviations: HR: hazard ratio; 95% CI: 95% confidence interval.

**Table S7. Evaluation of four biomarkers for progression of SCOPA-AUT**

|  | **Progression**  **(n=31)** | **Non-progression**  **(n=8)** | **HR**  **(95%CI)** | **p value** |
| --- | --- | --- | --- | --- |
| Age (y) | 68.5 ± 6.9 | 68.1 ± 10.8 | / | 0.89 |
| Sex, N |  | | | |
| Male | 20 | 6 | / | 0.65 |
| Female | 10 | 2 |  |  |
| ***CX3CL1*** |  | | | |
| Increased | 21 | 5 | 0.99  (0.94-1.04) | 0.63 |
| Decreased | 10 | 3 |  |  |
| ***IL-8*** |  | | | |
| Increased | 17 | 3 | 0.83  (0.38-1.81) | 0.63 |
| Decreased | 14 | 5 |  |  |
| ***CXCL12*** |  | | | |
| Increased | 8 | 1 | 1.14  (0.47-2.78) | 0.77 |
| Decreased | 23 | 7 |  |  |
| ***CCL15*** |  | | | |
| Increased | 25 | 6 | 1.82  (0.73-4.54) | 0.20 |
| Decreased | 6 | 2 |  |  |

Cox proportional hazards regression models were used to estimate HRs, with 95% CI adjusted for age and sex.

The bold emphasis in the table means *p* < 0.05.

Abbreviations: HR: hazard ratio; 95% CI: 95% confidence interval.

**Table S8. Evaluation of four biomarkers for progression of SS-16**

|  | **Progression**  **(n=29)** | **Non-progression**  **(n=10)** | **HR**  **(95%CI)** | **p value** |
| --- | --- | --- | --- | --- |
| Age (y) | 69.9 ± 6.4 | 64.3 ± 9.7 | / | 0.045 |
| Sex, N |  | | | |
| Male | 19 | 7 | / | 0.80 |
| Female | 10 | 3 |  |  |
| ***CX3CL1*** |  | | | |
| Increased | 19 | 7 | 0.89  (0.36-2.21) | 0.80 |
| Decreased | 10 | 3 |  |  |
| ***IL-8*** |  | | | |
| Increased | 15 | 5 | 0.73  (0.33-1.56) | 0.43 |
| Decreased | 14 | 5 |  |  |
| ***CXCL12*** |  | | | |
| Increased | 7 | 2 | 1.48  (0.59-3.74) | 0.41 |
| Decreased | 22 | 8 |  |  |
| ***CCL15*** |  | | | |
| Increased | 23 | 8 | 1.39  (0.55-3.53) | 0.49 |
| Decreased | 6 | 2 |  |  |

Cox proportional hazards regression models were used to estimate HRs, with 95% CI adjusted for age and sex.

The bold emphasis in the table means *p* < 0.05.

Abbreviations: HR: hazard ratio; 95% CI: 95% confidence interval.

**Table S9. Evaluation of four biomarkers for progression of RBDSQ**

|  | **Progression**  **(n=23)** | **Non-progression**  **(n=16)** | **HR**  **(95%CI)** | **p value** |
| --- | --- | --- | --- | --- |
| Age (y) | 68.0 ± 6.9 | 69.9 ± 9.9 | / | 0.50 |
| Sex, N |  | | | |
| Male | 14 | 12 | / | 0.36 |
| Female | 9 | 4 |  |  |
| ***CX3CL1*** |  | | | |
| Increased | 17 | 9 | 0.69  (0.21-2.22) | 0.53 |
| Decreased | 6 | 7 |  |  |
| ***IL-8*** |  | | | |
| Increased | 11 | 9 | 0.47  (0.19-1.16) | 0.10 |
| Decreased | 12 | 7 |  |  |
| ***CXCL12*** |  | | | |
| Increased | 5 | 5 | 0.64  (0.19-2.20) | 0.47 |
| Decreased | 19 | 11 |  |  |
| ***CCL15*** |  | | | |
| Increased | 18 | 13 | 1.66  (0.55-5.04) | 0.37 |
| Decreased | 5 | 3 |  |  |

Cox proportional hazards regression models were used to estimate HRs, with 95% CI adjusted for age and sex.

The bold emphasis in the table means *p* < 0.05.

Abbreviations: HR: hazard ratio; 95% CI: 95% confidence interval.
